# On the Beneficial Effects of the External Application of Opium in Inflammatory Diseases

**Published:** 1831-07

**Authors:** 


### OPIUM IN INFLAMMATION.

### *On the beneficial Effects of the External Application of Opium in Inflammatory Diseases.* By Dr. Bow.

In the work I lately published on the "Nature of Fever,"|| I endeavoured to draw attention to the external application of opium in that disease, as also in some inflammatory affections of the chest. I there stated my belief to be, that one of the

|| The work referred to was reviewed in our Journal for Jan. 1830.—Ed.

effects of opium so applied was to increase the contractile force of the arterial system; and, indeed, unless I am very much deceived, the experiments I detailed authorise me to say that such is its effect. However, impressed with this belief, it appeared to me that great benefit ought to be derived from its application in every case of congestion of the capillaries; and, being already satisfied with its operation where such congestion was general, and consequently slight, as in fever, I determined to trust to it also when local, as in inflammation. I will now, with your permission, transcribe a few cases from my note-book, in which the success of the plan exceeded my anticipations.

CASE I. 14th February, 1831. — Darling, aged nine months, was reported at noon, by Mr. TENDER,* to be labouring under bronchitis. I immediately proceeded with him to the patient; found the breathing difficult, the inspirations being short and frequent, accompanied with a wheezing noise; the face very pale, the lips having a purple tinge; skin exceedingly hot; hoarseness of the voice when the child cried; pulse rapid. The child had been ill for some days, but the mother, thinking that nothing ailed it except a common cold, did not become alarmed until this morning. Nothing had been prescribed before I saw it. Two grains of calomel were given, and the breast and back rubbed with rather more than a drachm of opiate liniment.†

The calomel was rejected from the stomach almost as soon as taken. After the application of the liniment, the child fell into a sound sleep: at two o'clock he awoke, and sucked greedily; and at our visit, a little after two, we found him in a profuse perspiration, the voice perfectly free from hoarseness, and the breathing comparatively easy. At four o'clock, the breathing had again become difficult, but, in degree, nothing equal to what it was in the morning: the calomel and liniment were repeated. At six o'clock, the child seemed calm and contented; the eyes were sprightly, and some colour had returned to the cheeks. A portion of liniment was left with the mother, with orders to apply it, should the breathing again become hurried.

15th February. About three o'clock this morning the liniment was applied, as the mother thought the state of the breathing required it. At our visit at ten A.M., we found that the bowels had been twice moved; the effect of the calomel. The child seemed quite well, and therefore nothing was prescribed. At seven P.M. the child continued well. At the request of the mother, a portion of the liniment was left, to be applied if required.

16th. The liniment was not applied. Cured.

* Resident surgeon, Alnwick Dispensary.

† ℞. Opii ʒi.; Linim. Camph. c. ℥i. Digere per dies aliquot et effunde linimentum.

II. 16th February, 1831. E. Race, aged ten months. Visited this child today, in company with Mr. Fender, at eleven A.M.: there was great heat of skin; restlestness; difficult breathing, with wheezing; paleness of the face; purple hue of the lips; lividity around the mouth; pulse 160, or more, (it was difficult to count it.) The mother said that the breathing had been so difficult at intervals during the last two nights, that she thought the child would have expired. A drachm and a half of liniment was ordered, which Mr. Fender applied before we left the house.

At one o'clock, the child slept soundly; pulse 120. The respirations, which, at our first visit, could not be numbered, owing to the restlessness of the little patient, were now ninety-six in the minute; skin comparatively cool. At three o'clock, the child was still asleep; pulse 110; respirations eighty: liniment repeated.

At ten P.M., pulse ninety-six; respirations sixty-eight: liniment repeated.

17th. The child had a good night, and is now apparently well. As she could not be kept sufficiently quiet, neither the pulse nor respirations could be numbered. Cured.

III. 23d February, 1831. — Dixon, aged eight months. I saw this child at six this evening: it has had catarrhal symptoms since the 20th instant. I found the breathing hurried; the skin hot; face pale; great restlessness; pulse rapid. A drachm and a half of the liniment was prescribed.

24th. After the application of the liniment, the child slept for some hours: towards midnight, it awoke in a profuse perspiration. About break of day, it again fell asleep, and continued to sleep soundly until eight o'clock A.M. I saw it at half-past nine, and, as it appeared perfectly well, I did not prescribe for it.

At three P.M. I was again sent for, and was told that, towards noon, the child (as the mother expressed it) "got quite knocked," since which it had gradually got worse. I found the skin extremely hot, and the breathing hurried, with wheezing. The child was quiet and drowsy, so that the pulse and respirations were easily reckoned; the former 160, the latter 84. I prescribed two grains of calomel, and a drachm and a half of liniment.

Ten P.M. Shortly after the liniment was applied, the child became lively and playful. At present he is in a calm sleep; heat of skin less; pulse 120; respiration seventy, no wheezing. Repeat the friction, and one grain of calomel.

25th, ten A.M. The child passed a good night; the bowels have been twice moved this morning; appears lively, and apparently quite well. Took my leave.

IV. 14th February, 1831. M. Y., aged three years six months, for some days past has had stuffing about the chest, cough, burning skin, and great restlessness in bed during night. I saw her for the first time at nine P.M. this evening: the breathing was difficult, with wheezing; the face flushed; tongue foul; pulse 120. Two grains of calomel were ordered.

15th, one P.M. The bowels have been moved: the difficulty of breathing continues; skin hot; face still flushed; pulse about 130. A drachm and a half of the liniment to be repeated at bedtime.

16th. At my visit this morning, the child was at play in a neighbour's house: she had a good night, and is reported quite well.

I might detail many cases, somewhat similar to the last, which were all cured, as if by charm, by one or two applications of the liniment; but as I look on them as cases of catarrh only, I shall rest satisfied, trusting the above may induce some of your readers to adopt the practice. My object is to weaken, if possible, the faith which is now so injuriously reposed in bloodletting for the cure of inflammations in early age. In bronchitis, for example, the great mortality under the practice most approved of, shews that bleeding, general or local, is not infallible; and that it is too often mischievous in its immediate effects, that keen investigator, Dr. Marshall Hall, amongst others, has sufficiently demonstrated. If we do succeed in subduing the disease by bloodletting, we have still the remote unhappy effects to dread; for it, not unfrequently, changes altogether the temperament of the individual. To this effect no one can be blind: every day we meet with, in families generally healthy and robust, one or two delicate members, who are liable to colds and rheumatism upon every atmospheric change, peculiarly obnoxious to every disorder which may rage, and slow in recovering from the most trifling. On inquiry, we learn that these individuals were healthy and strong until attacked by croup, chincough, or some other disease, for the removal of which copious depletion was thought necessary.

I believe there are few who are not converts to the theory which makes inflammation to consist in debility of the capillaries of the part inflamed: still this theory, so generally embraced, has in no way modified the plan of treatment; for the same mode is advised, and is had recourse to, as when we believed the disease to consist in excitement of the same vessels. This is owing to half measures, to moderate reform: the advocates of the theory have taken excitement from the smaller vessels, but they have permitted the larger to retain it; consequently, to *moderate* the *excitement* of the sanguiferous system is the chief aim, and bloodletting is advised as the most powerful means. But the apparently increased action of the larger arteries, mistaken for, or miscalled *excitement*, depends upon loss of contractile power: therefore it is our business to invigorate the arterial system throughout, so that the arteries may be enabled to contract forcibly on their contents, and thus relieve themselves: not to debilitate them further and further, until they become like inanimate tubes, without power to assist in the circulation.*

If I had effected a cure, in the cases given, by the usual means, leeching, blistering, &c., I should have debilitated my patients so such a degree that my attendance could not have been dispensed with for many days; for, in reality, by such means we create a new disease: as it was, as soon as the inflammation was subdued, all care was over, for health was re-established, and strength immediately restored; in fact, there was no convalescent period.

Observe, in the case of Race, how rapidly and steadily the pulse diminished in frequency: in twelve hours it was brought from upwards of 160, to its natural state, ninety-six. This case I regarded as hopeless; and, that the opiate friction might have a fair trial, I refrained from administering any thing internally. Calomel, however, in tolerably large doses, ought never to be dispensed with, for it acts speedily in exciting the capillary vessels to contract.

In the following case I was not so successful, and I see no reason why I should not impute the failure to the too great detraction of blood.

19th February, 1831. — Hay, aged nine months. At two P.M. I saw this child for the first time. About three quarters of an hour before I saw it, two leeches had been applied to the breast, from the bites of which the blood still flowed; face very pale; lips of a deep purple; lividity around the mouth; pulse rapid and fluttering; respirations ninety, irregular; pupil dilated. There was nothing to indicate life but the motion of the chest and wheezing noise. The child has been ill since the evening of the 15th, and yesterday there were applied to the breast a blister and two leeches. The bowels were open, evidently from the operation of calomel. I ordered the bleeding to be checked as soon as possible, and a drachm and a half of the liniment to be rubbed on the back and bowels: the blistered surface preventing its application to the chest.

At five o'clock P.M. the respirations were sixty-four; wheezing

* Perhaps it may be difficult to conceive that the heart and arteries should, during febrile excitement, be deficient in contractility, or the power with which they contract, seeing that the vascular commotion is frequently very violent: nevertheless, it is true; and he who would most effectually quell such commotion, with his patient's welfare in view, will attempt it by invigorating the arterial system, which is to be accomplished by obviating the preternatural determination of nervous influence to the surface of the body. ("Notions of the Nature of Fever and of Nervous Action; by W. Forrester Bow, M.D. &c." page 99.)

almost inaudible. Although the child played with my watchchain, it seemed so exhausted as to require a teaspoonful of sherry. The liniment was repeated.

At nine P.M. it continued easier. Liniment repeated.

20th, ten A.M. Had a tolerable night: the wheezing at present is considerable, but the attendants say it is only occasionally so. Repeat the liniment.

Six P.M. Has taken a good deal of nourishment, and slept calmly at intervals, during the day.

21st, nine A.M. Has passed a tolerable night. There is expectoration, but, as the child swallows it, it cannot be examined; wheezing at intervals. Liniment.

Four P.M. The child has taken food repeatedly during the day, and was calm and cheerful all the forenoon. Towards three o'clock, the breathing became oppressed, (the effect of too much food, I presume,) and, as I was in the country, some one, unfortunately, advised a warm bath, followed up by heated blankets. The child is now in a profuse perspiration.

Eight P.M. Evidently worse; pulse fluttering and scarcely perceptible. A teaspoonful of sherry occasionally.

22d, four A.M. Apparently sinking fast. Rubbed with two drachms of laudanum; a teaspoonful of sherry every hour.

Ten A.M. More lively, and attentive to the motions of the attendants. Continue the sherry.

Ten P.M. Is still lively: has taken food during the day.

24th. The child was better and worse during yesterday. Died this day.

The following case of croup was one of the most formidable I ever witnessed.

4th March, 1831. William Douglas, aged two years six months, has had catarrhal symptoms for some days past: yesterday morning, his breathing was heavy and cough croupy; towards evening, he appeared worse; and, about eleven o'clock last night, the respiration became more difficult and sonorous. I saw him for the first time this morning, at three A.M.: the breathing was very difficult, and the sound emitted so loud that I heard it on approaching the outer door of the house; he was extremely restless; skin hot; face flushed; pulse frequent. An emetic was given, and, after he had vomited, the breast and back were rubbed with two drachms of liniment. In half an hour, he was so far relieved as to breathe with less difficulty: the respiration was not sonorous unless when he struggled, which he did once or twice to get down from his nurse's knee. Two grains of calomel were given, and about half a drachm of lard, containing five grains of opium, was rubbed in on his limbs and back. Soon after he was rubbed, he became lively and playful, and then fell into a sound sleep, in which I found him at six o'clock. He perspired freely: but as his breathing was thick, though not sonorous, I awoke him, and administered two grains of calomel, with a quarter of a grain of opium.

I desired they would call me at half-past seven: the report then was, that he slept quietly, and breathed easily.

At nine o'clock I saw him, and was happy to observe that the respiration was almost natural: he certainly breathed with some difficulty, but, as there was no restlessness, no heat of skin, and a pulse not at all frequent, had I seen him then for the first time, I should have thought nothing of it. Two grains of calomel.

At eleven o'clock, the child was playing about the room, but, as the state of his breathing did not yet satisfy me, I ordered his neck and breast to be rubbed with two drachms of laudanum; and, as I was forced to proceed to some distance, I prescribed two grains of calomel every two hours.

At eight P.M. I found him in a sound sleep; respiration perfectly free. Three of the powders had been given; the bowels had been twice moved. His nurse told me that, soon after he had been rubbed with the laudanum, he coughed a good deal, and expectorated some thickish matter: from that moment, she said, he was quite himself. The expectorated matter she did not preserve. Nothing prescribed.

5th, noon. Continues well; the bowels have been moved since last visit.

6th, two P.M. He is romping about the house, apparently in perfect health.

This case requires little comment: there can be no doubt that, before I saw the patient, exudation of lymph, to a certain extent, must have taken place; which accounts for the respiration continuing troublesome after the inflammatory action had been arrested by the first application of the liniment. If I had trusted to bloodletting, I must have taken a large quantity; for the symptoms were so urgent, that a small bleeding would have been of no use; and, had I succeeded, would my little patient have been able to resume his play in a few hours, as if nothing had happened? I suspect he would have been so debilitated, before the inflammation was subdued, that he could not have had power to expectorate, and thus would have died from suffocation, as many have.

I made use of laudanum in the above case, because I could get no more of the liniment. I am not at all persuaded, however, that the liniment I use is the best application. I think some preparation of belladonna might still be better. The plan I propose has one thing to recommend it, and which I am certain will ensure it a trial, even by those most devoted to the lancet: it is, that the good effects very soon become conspicuous. Let them wait half an hour, and if in that time no relief be afforded, little harm can accrue from the short time lost.

*Alnwick.*

